# ALK-negative inflammatory myofibroblastic tumor with an undetermined differentiation direction: A case report and review of the literature

**DOI:** 10.1097/MD.0000000000047278

**Published:** 2026-01-16

**Authors:** Huihui Guo, Hongjing Wang

**Affiliations:** a Department of Gynecology and Obstetrics, West China Second University Hospital, Sichuan University, Chengdu, Sichuan Province, P.R. China; b Key Laboratory of Birth Defects and Related Diseases of Women and Children (Sichuan University), Ministry of Education, Chengdu, Sichuan Province, P.R. China.

**Keywords:** anaplastic lymphoma kinase, broad ligament, inflammatory myofibroblastic tumor, mesenchymal neoplasms, molecular profiling, rare tumor, targeted therapy

## Abstract

**Rationale:**

Inflammatory myofibroblastic tumor (IMT) is a rare mesenchymal neoplasm that can arise in various anatomical locations, while IMT originating from the broad ligament is exceptionally rare. Anaplastic lymphoma kinase (ALK) is a highly specific diagnostic biomarker for IMT. However, no standardized therapeutic algorithm has been established for ALK-negative IMT, largely due to the unclear risk of distant metastasis and recurrence. In cases with undefined tumor differentiation and no detectable molecular targets, close surveillance and individualized therapeutic strategies should be guided by the tumor’s biological behavior and patient-specific risk factors.

**Patient concerns:**

We present the case of a 21-year-old unmarried, nulliparous woman who reported a palpable abdominal mass, ultimately found to originate from the broad ligament.

**Diagnoses:**

Exploratory laparotomy revealed a mass arising from the posterior leaf of the broad ligament along the posterior uterine wall. Histopathological analysis favored a diagnosis of ALK-negative IMT, although the origin and differentiation trajectory of the tumor remained undetermined.

**Interventions:**

The patient did not undergo any adjuvant therapy, such as chemotherapy. Instead, a regimen of routine follow-up was implemented to monitor for disease progression or recurrence.

**Outcomes:**

At the 10-month follow-up, no clinical or radiological signs of recurrence or malignant transformation were observed.

**Lessons:**

To date, surgical resection remains the mainstay of treatment for IMT. For ALK-negative IMT cases, molecular profiling to identify targetable genomic alterations may assist in selecting individualized targeted therapies.

## 1. Introduction

Inflammatory myofibroblastic tumor (IMT) is a rare mesenchymal neoplasm characterized by spindle-cell proliferation accompanied by varying degrees of inflammatory cell infiltration. Initially reported as originating in the lungs, IMT was first described as an inflammatory pseudotumor of the pulmonary region. An inflammatory pseudotumor is a heterogeneous inflammatory mass, histologically defined by myofibroblast proliferation and chronic inflammatory cell infiltration. It typically follows a benign course. However, due to its mass-like morphology and radiological features, it is often misdiagnosed as a malignant neoplasm. According to existing literature, IMT can arise in various extrapulmonary sites, although its presence in the broad ligament of the uterus is extremely rare. The most common sites include the abdominopelvic region, pulmonary parenchyma, and retroperitoneal space.^[1]^ IMT is now recognized as a distinct pathological entity. The World Health Organization classifies IMT as a fibroblastic/myofibroblastic neoplasm with intermediate biological potential.

The etiology of IMTs remains unclear. Potential predisposing factors include trauma, bacterial or Epstein–Barr virus infections,^[2]^ immune dysregulation,^[3]^ chromosomal abnormalities, aberrant tissue repair, major surgical interventions,^[4]^ and the spread of inflammation.

IMT presents with nonspecific clinical features, and preoperative diagnosis remains challenging. Therefore, histopathological confirmation via biopsy is essential. Histologically, IMT consists of neoplastic myofibroblastic spindle cells accompanied by infiltration of inflammatory cells, including lymphocytes and plasma cells.^[5]^ Complete surgical resection is the standard treatment for localized IMT. However, for advanced-stage or unresectable IMT, there is currently no internationally accepted standard treatment protocol. Advances in molecular diagnostic techniques and improved tumor classification have made targeted therapy for IMT both a promising option and a significant challenge.

Anaplastic lymphoma kinase (ALK) is a receptor tyrosine kinase, and in a subset of IMT cases, rearrangements of the ALK gene – located on chromosome 2p23 – have been detected using fluorescence in situ hybridization (FISH) or polymerase chain reaction (PCR).^[6]^ ALK positivity serves as a specific biomarker for distinguishing IMT from other neoplasms; however, ALK negativity does not preclude the diagnosis. In ALK-negative cases, the underlying genetic mechanisms remain unclear, and treatment options are limited, although complete surgical resection remains the primary recommendation. To date, existing research has only partially characterized ALK-negative cases, and the investigation into potential fusion genes remains in its early stages. With the rapid advancement of molecular testing technologies, novel molecular subtypes are continuously being identified. This progress has significantly improved the diagnostic classification and prognostic assessment of mesenchymal neoplasms. The spectrum of undifferentiated tumors has progressively narrowed. Furthermore, unlike conventional treatment approaches, this evolving understanding has made targeted therapy a feasible option.

## 2. Case presentation

**Figure 1.**
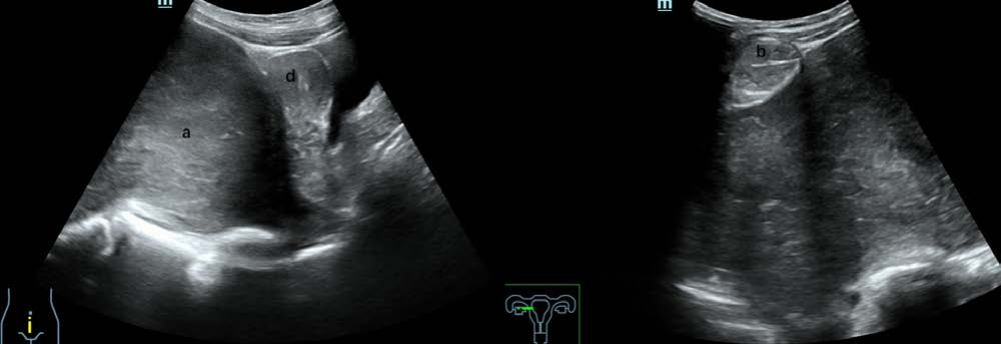
A heterogeneous mass (labeled a) was visualized posterior to the uterus (labeled d). Adjacent to the right ovary, a cystic-solid mixed space-occupying lesion (labeled b) was identified. Both lesions showed well-defined boundaries and relatively regular configurations.

**Figure 2.**
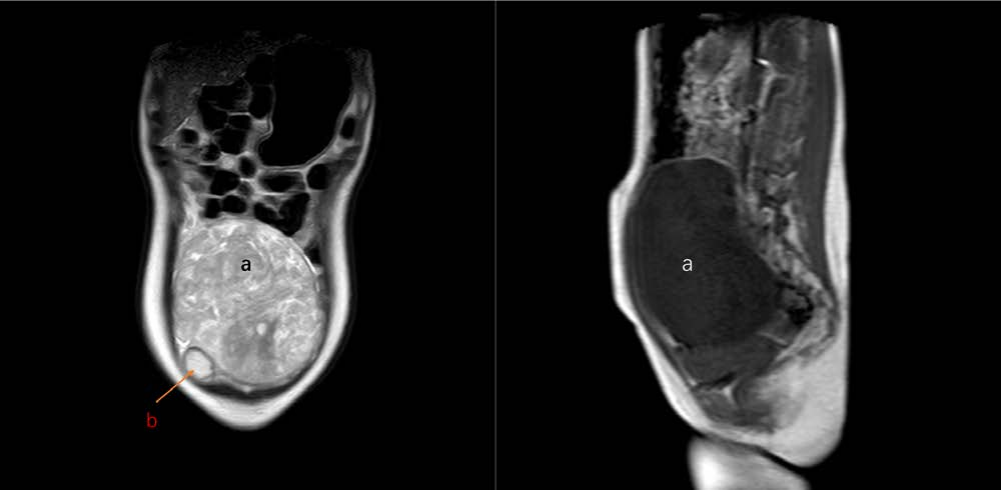
Abdominopelvic enhanced MRI revealed a large mass (a) with heterogeneous enhancement. The posterior margin of mass (a) is closely adjacent to the presacral region and causes significant compression of the sigmoid colon; its superior margin extends to the level of the fourth lumbar vertebra (L4), and inferior margin is closely adherent to the posterior uterine wall. Mass (b) is predominantly cystic, unilocular, and contains fatty components.

A 21-year-old unmarried, nulliparous woman with no significant family history of neoplastic diseases presented voluntarily with a palpable abdominal mass and a history of constipation lasting several weeks. Before seeking medical attention, she had experienced right-sided abdominal distension and pain, accompanied by mild nausea and urinary frequency. She subsequently underwent color Doppler ultrasound (Fig. 1) and contrast-enhanced magnetic resonance imaging (MRI) (Fig. 2). Both imaging studies revealed a large, subcircular pelvic mass with well-defined borders located posterior to the uterus, with an undetermined origin. Additionally, a mixed cystic-solid lesion measuring approximately 4 cm was identified adjacent to the primary mass, although the relationship between the 2 lesions remains unclear. Laboratory tests revealed normal serum levels of cancer antigen 125 (CA125), carcinoembryonic antigen (CEA), and squamous cell carcinoma antigen (SCC-Ag).

**Figure 3.**
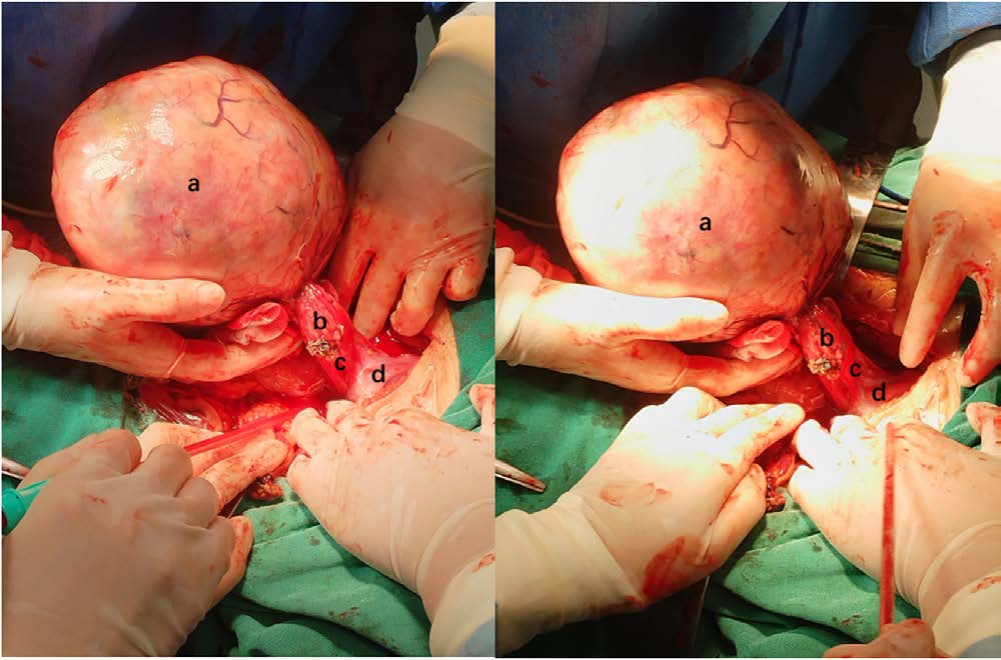
Mass (a) exhibited a smooth surface and well-defined margins. To the right of mass (a) is mass (b), which is predominantly cystic and arises from the posterior lobe of the broad ligament (labeled c) on the posterior uterine wall (labeled d), with subserosal protrusion.

Based on these findings, the gynecologic oncology tumor board recommended resection of the pelvic mass due to suspected malignancy. On July 7, 2024, the patient underwent preoperative preparation followed by an exploratory laparotomy. Intraoperatively, a solid pelvic mass (designated as mass a), measuring 17 × 14 × 11 cm, was identified, with a smooth, whitish surface. Mass a was clearly demarcated from the uterus and bilateral adnexa. A cystic-solid mass, approximately 4 cm in diameter (designated as mass b), was identified on the right side of mass a. Mass b originated from the posterior leaf of the broad ligament, adjacent to the posterior uterine wall, and protruded beneath the serosal membrane (Fig. 3). Intraoperative frozen section analysis revealed that mass b consisted of fibroadipose tissue with accompanying inflammatory cell infiltration. Mass a was identified as a mesenchymal tumor with necrotic features. As the intraoperative frozen sections did not reveal malignant components in either mass, no additional extensive surgical procedures were undertaken.

The patient had an uneventful recovery and was discharged on postoperative day 5. Postoperative abdominopelvic contrast-enhanced computed tomography (CT) and tumor marker tests showed no evidence of malignancy. At the 10-month follow-up, the patient remained under regular surveillance, with no evidence of tumor recurrence.

### 2.1. Pathology and microscopy

**Figure 4.**
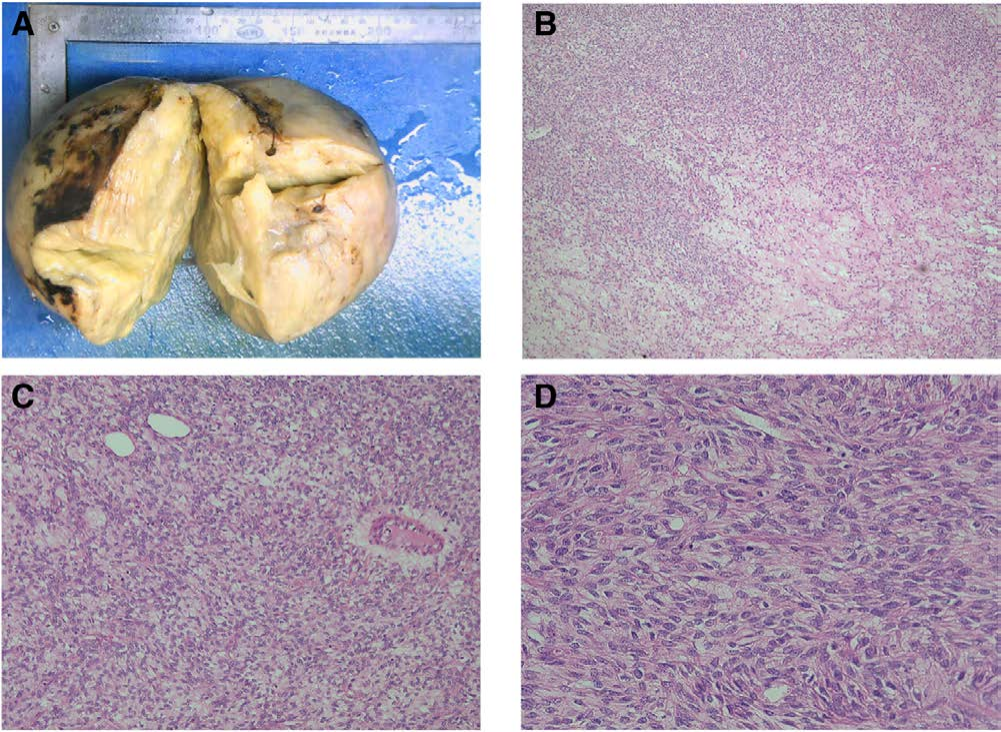
Macroscopically, the mass (a) manifested as fibroid-like tissue with a firm texture (A). Pathological evaluation of the tumor, the mass (a) was characterized by spindle-cell proliferation. Hematoxylin–eosin staining, ×40 (B); ×100 (C); ×200 (D).

Macroscopically, mass a consisted of fibroid-like tissue with a firm consistency, with areas of focal hemorrhage and necrosis. Histologically, mass a was diagnosed as a spindle-cell tumor with mild atypia; mitotic figures were readily identified (>10 per high-power field), and focal necrosis was observed (Fig. 4).

### 2.2. Immunohistochemistry and molecular detection technologies

**Figure 5.**
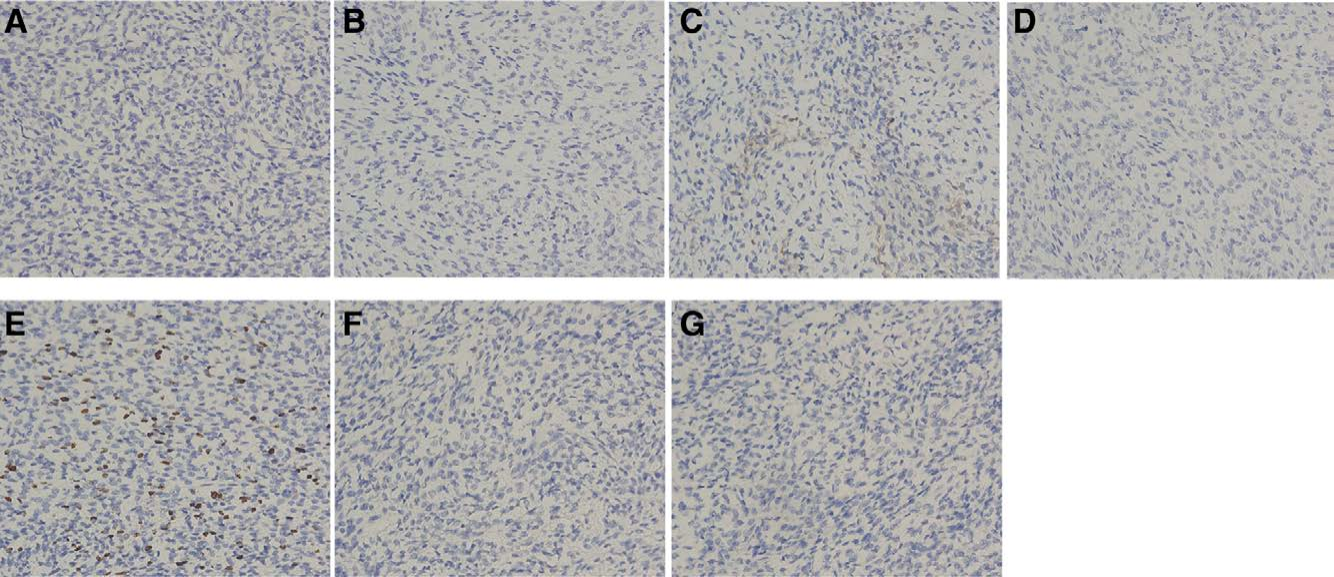
Immunohistochemical staining of the tumor (×200): mass (a) cells were completely negative for anaplastic lymphoma kinase (ALK) (A), caldesmon (B), desmin (D), S-100 (F), and STAT6 (G); focal positivity for CD10 was observed (C); the Ki-67 proliferation index was approximately 20% in the hotspot areas (Panel E).

Immunohistochemical staining revealed focal positivity for CD10, with a Ki-67 proliferation index of approximately 20% in hotspot areas; all other markers were negative (Fig. 5). Based on the immunohistochemical profile, the findings were most consistent with a mesenchymal neoplasm.

**Figure 6.**
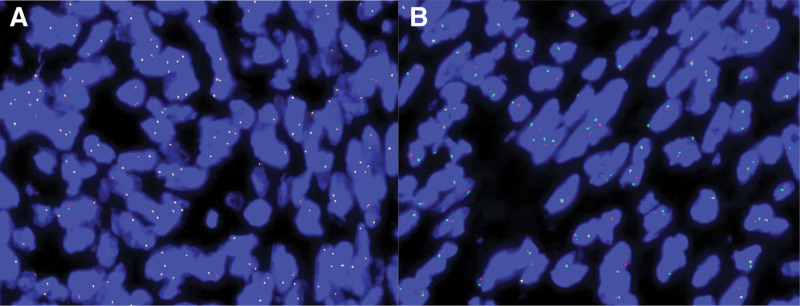
Dual-color break-apart FISH was performed on 5-μm sections. A quantity of 100 tumor cells was enumerated. No meaningful translocation of the BCOR gene (Xp11.4) was detected (A). No amplification of the MDM2 gene (12q15) was detected (B). FISH = fluorescence in situ hybridization.

**Figure 7.**
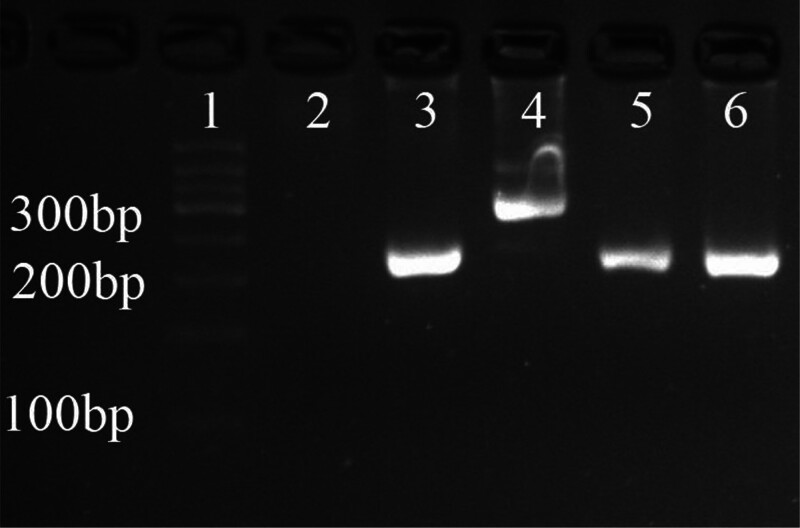
PCR testing for BCOR-ITD mutation. (1) Marker; (2) blank control; (3) negative control; (4) positive control; (6) specimen. BCOR-ITD = BCOR-internal tandem duplication, PCR = polymerase chain reaction.

A joint pathological consultation was conducted with the Department of Pathology at West China Hospital. Two pathologists from West China Hospital independently reviewed the pathological slides. Microscopically, tumor cells in mass a exhibited plump spindle-shaped morphology, mild-to-moderate atypia, abundant thin-walled small vessels, and multifocal necrosis. Mitotic figures were readily observed at a rate of 5 to 7 per 10 high-power fields. In addition, variable lymphocytic infiltration was observed in different regions. The characteristic marker ALK showed negative immunoreactivity. FISH revealed no clinically significant BCOR gene translocation or MDM2 gene amplification (Fig. 6). PCR also failed to detect BCOR-internal tandem duplication (BCOR-ITD) (Fig. 7). Given that genetic testing revealed no evidence of tumor origin, differentiation pathway, or relevant genetic alterations – and considering the histological findings of mild-to-moderate atypia, readily identifiable mitotic figures, elevated proliferative index, and necrosis – pathologists currently favor a diagnosis of either an ALK-negative IMT or a rare low-grade malignant/indolent mesenchymal neoplasm with uncertain differentiation and intratumoral thrombosis.

Finally, next-generation sequencing (NGS) was performed on formalin-fixed, paraffin-embedded (FFPE) tissue specimens. No clinically relevant Class I, II, or III variants were detected, based on evaluation criteria from the 2017 *Guidelines for the Interpretation and Reporting of Sequence Variants in Cancer*, jointly issued by the *American Society of Clinical Oncology*, the Association for *Molecular Pathology*, and the *College of American Pathologists*.

## 3. Discussion

The uniqueness of this case lies in the tumor’s origin from the broad ligament of the uterus, an exceedingly rare anatomical site. Despite comprehensive diagnostic efforts, both ALK immunohistochemistry and FISH results were negative, leaving the tumor’s lineage and differentiation uncertain. Due to the rarity of relevant literature, this report focuses on uterine IMTs (UIMT), aiming to provide a comprehensive discussion of their clinicopathological characteristics.

UIMT is a rare neoplasm of the female genital tract with uncertain malignant potential. Histologically, it is characterized by fibroblastic spindle-shaped cells, variable degrees of myxoid stroma, and lymphoplasmacytic inflammatory infiltration.^[7]^ It can occur at any age in females; however, it is most commonly observed in women of reproductive age. Rare cases have been reported in pediatric patients,^[8,9]^ and it has also been detected in pregnant women.^[10,11]^ In elderly patients, IgG4-related disease should be considered in the differential diagnosis of IMT.^[1]^ Among female reproductive organs, the uterine corpus is the most common site of IMT. Specifically, IMT accounts for only 0.1% of all uterine leiomyomas. However, the incidence rises to 10% among pregnant women and up to 14% in uterine leiomyomas of uncertain malignant potential.^[12]^

The clinical presentation of UIMT is often heterogeneous, primarily depending on the tumor’s size and anatomical location.^[8]^ The tumor is typically located within the uterine myometrium but may also arise in the submucosal layer, presenting as a polypoid lesion. Rarely, it has been reported in the uterine cervix. However, reports of UIMT arising from the uterine serosa remain exceedingly rare.^[7,13–15]^ Clinical manifestations may include abnormal uterine bleeding, dysmenorrhea, uterine leiomyomas, or palpable pelvic masses. Moreover, a substantial proportion of patients remain asymptomatic. Clinical features of IMT, whether in the female reproductive tract or other anatomical sites, are nonspecific across both ALK-negative and ALK-positive subtypes, making preoperative differentiation nearly impossible. Diagnosis is generally established based on histopathological examination after surgical excision of the lesion. Preoperative diagnosis is challenging, with high rates of misdiagnosis and missed diagnosis, posing a significant clinical dilemma. It is particularly difficult for gynecologists to recognize this rare entity prior to surgery or biopsy.^[9]^ As a result, the true incidence of UIMT may be significantly underestimated. In the present case, the patient had a prior history of menstrual irregularities associated with polycystic ovary syndrome. She subsequently developed symptoms related to tumor compression. Notably, all clinical symptoms in this case were nonspecific.

Preoperative imaging assessment is now widely employed. Contrast-enhanced CT and positron emission tomography–CT (PET–CT) are commonly used to evaluate tumor invasion and metastasis. In contrast, MRI more effectively assesses soft-tissue resolution and avoids radiation exposure.^[8,9,16]^ MRI findings of IMT may resemble those of infiltrative, fibrotic, or highly cellular lesions. Color Doppler ultrasound and MRI examinations performed at our hospital both revealed a heterogeneous pelvic mass. Unlike other malignant tumors, this mass displayed regular margins and clear demarcation from adjacent tissues.

In pathological diagnosis, UIMT must be differentiated from leiomyoma, sarcoma, endometrial stromal tumor, fibroleiomyomatosis, and desmoid-type fibromatosis.^[17–20]^ UIMT differs from uterine leiomyoma in both texture and morphology. It typically appears softer and gelatinous, with either well-defined or irregular margins. Histologically, UIMT can be classified into myxoid/vascular, densely packed spindle-cell, or hypocellular patterns, often accompanied by variable amounts of chronic inflammatory infiltrates. A definitive diagnosis requires integration of histological features, immunohistochemical profiles, and molecular testing, including FISH or NGS.

ALK is negative in normal uterine tissues, and to date, no ALK rearrangements have been detected in uterine leiomyomas. ALK immunohistochemistry (IHC), FISH, and RNA sequencing have demonstrated high specificity in the diagnosis of IMT. Both FISH and RNA sequencing are effective for confirming the presence of ALK rearrangements; however, each technique has inherent limitations. Currently, diagnosis primarily relies on the integration of histopathological examination and immunohistochemistry. IMT typically expresses smooth muscle markers (desmin, SMA, caldesmon, transgelin) and stromal markers (CD10, IFITM1), which can lead to misdiagnosis as leiomyoma or endometrial stromal tumor. IMT is often positive for estrogen receptor and progesterone receptor, may show weak BCOR expression, and is typically negative for keratin.^[7,13,21–23]^ Approximately 50% to 60% of IMTs harbor ALK gene rearrangements on chromosome 2p23, resulting in constitutive tyrosine kinase activation.^[24,25]^ To date, multiple ALK fusion partners have been identified, including TPM3, TPM4, CLTC, and ATIC.^[6,26,27]^ In ALK-negative IMTs, it is essential to screen for other common fusion driver genes using FISH, PCR, or NGS to identify actionable therapeutic targets, which are critical for guiding subsequent treatment decisions. Concurrently, comprehensive clinical assessment is necessary to determine tumor behavior and guide decisions regarding surgical versus pharmacological intervention. In some ALK-negative cases, alternative genetic alterations such as RET, ROS1, or NTRK3 fusions may be present.^[28,29]^ These fusions may serve as oncogenic drivers in the pathogenesis of IMT. Notably, TIMP3-ALK and THBS1-ALK fusions are enriched in pregnancy-associated uterine IMTs.^[14,30–33]^ The RANBP2-ALK fusion has been identified in epithelioid inflammatory myofibroblastic sarcoma (eIMS) and is associated with aggressive clinical behavior and poor prognosis.^[34,35]^ Therefore, in ALK-negative cases, additional FISH or RNA sequencing is essential for diagnostic clarification. Several studies suggest that sequencing technologies may offer greater diagnostic utility. These techniques can be repurposed to detect gene fusions, mutations, and resistance variants within tyrosine kinase domains.^[36,37]^ In the present case, both ALK immunohistochemistry and FISH yielded negative results. Furthermore, NGS of the tumor revealed no significant genetic alterations. NGS analysis of the tumor did not identify any gene fusions or mutations. During follow-up, no evidence of recurrence or distant metastasis was observed.

For women without fertility requirements, total hysterectomy is a viable treatment option. For women of reproductive age, lesion excision combined with careful postoperative follow-up may be considered. Studies have shown no significant difference in prognosis between lesion excision and total hysterectomy.^[38]^ In IMTs located at other anatomical sites, recurrence rates remain low even after conservative surgical management.^[39,40]^ However, tumors with otherwise bland histological features may still recur.^[14,21]^ Positive surgical margins are the most common cause of local recurrence. Regarding distant metastasis, the most frequently affected sites are the lungs and brain, followed by the liver and bones.^[41]^ Some studies suggest that IMT recurrence rates may be associated with the tumor’s primary anatomical location. According to the 2020 World Health Organization classification, tumors larger than 7 cm, with moderate to severe atypia, high mitotic activity, necrosis, or lymphovascular invasion, are associated with malignant behavior.^[42]^ These features may contribute to recurrence or progression to invasive IMT, resulting in poor prognosis.^[30]^ In cases where surgical resection is not feasible due to anatomical constraints or multiple comorbidities, the optimal treatment strategy remains controversial. Nonetheless, adjuvant radiotherapy and chemotherapy should be considered as potential treatment options.^[43]^ Some evidence suggests that ALK-negative IMTs may have a greater propensity for metastasis.^[1,41,44]^ Cheryl M. et al reported a correlation between ALK negativity and local recurrence, but not with distant metastasis.^[41]^ However, the prognostic significance of ALK negativity in IMT remains unclear.

Currently, there is clinical interest in developing specific biomarkers or detection tools capable of predicting tumor invasiveness. Several studies have demonstrated associations between abnormal p16 expression, CDKN2A deletion, TP53 mutations, and malignant behavior.^[21,22,45]^ In this case, although no definitive genetic alterations were identified, the pathology consultation suggested a high likelihood of multifocal tumor origin. During postoperative follow-up, no signs of malignancy or tumors at other anatomical sites were observed. Nevertheless, the patient will continue to undergo close and comprehensive follow-up.

Due to the inherent inflammatory nature of IMT, anti-inflammatory agents such as corticosteroids and nonsteroidal anti-inflammatory drugs (NSAIDs) were among the earliest treatments used. A clinical study by Chaves et al reported that NSAIDs were effective in 10 of 11 ALK-negative IMT cases, resulting in significant disease remission.^[46]^ Hidehiro et al reported the first case of IMT that was both ALK-negative and IgG4-negative. The patient presented primarily with chronic respiratory inflammation, and clinical improvement was achieved following treatment with clindamycin, a macrolide antibiotic.^[47]^ Chemotherapy may be considered for rapidly progressive, recurrent, unresectable, or metastatic IMT, or when surgery is contraindicated due to poor performance status. In surgically unresectable IMT, the methotrexate/vinblastine (MTX/VBL) chemotherapy regimen may serve as an effective alternative.^[37]^ However, no standardized treatment protocol exists, and current regimens are largely based on clinical experience or small-scale studies; their efficacy remains to be clearly established.

According to the *NCCN Guidelines* (Version 2.2023), ALK inhibitors – crizotinib, ceritinib, brigatinib, lorlatinib, and alectinib – are approved as first-line therapies for IMT cases with ALK rearrangements.^[48]^ Several studies have reported that ALK inhibitors can be used to treat recurrent or refractory IMT. No definitive treatment regimen has been established for ALK-negative patients. In cases with ROS1 fusions, ROS1 inhibitors (e.g., crizotinib) may be considered, as clinical studies have shown some efficacy in ROS1 fusion-positive IMT. Hornick et al reported that pediatric patients with pulmonary IMT harboring TGF-ROS1 fusions benefited from crizotinib therapy (250 mg).^[49]^ If NTRK fusions are detected, NTRK inhibitors such as larotrectinib and entrectinib may be used. These agents are approved for the treatment of NTRK fusion-positive solid tumors, including IMT.^[50–52]^ Nevertheless, potential therapeutic benefits must be carefully evaluated on a case-by-case basis.^[53–56]^ Numerous ALK inhibitors are currently undergoing clinical trials. Their clinical efficacy and potential adverse effects have yet to be clearly defined. Therefore, large-scale clinical studies are urgently needed. In pregnant women, tyrosine kinase inhibitors may represent a novel therapeutic option.^[57]^ Furthermore, high PD-L1 expression in ALK-negative IMT highlights the potential of immune checkpoint pathways as therapeutic targets in recurrent or refractory disease.^[58,59]^ Targeted therapies generally provide better efficacy and a more favorable safety profile than conventional chemotherapy, making them preferred options for precision treatment in ALK-negative IMT. Table 1 summarizes reported cases of locally recurrent and metastatic IMT published in PubMed between 2020 and 2025, along with associated treatment strategies.^[60–69]^

**Table 1:** Cases of local recurrence and distant metastasis from 2020 to 2025.

Author [ref]	Gender	Age	Primary site	Metastatic site	Local recurrence	ALK	Treatment	Gene sequencing	Aggressive behavior
T. C. Hou et al^[60]^	Female	69	Bulky retrosternal mass	Neck lymph node	+	−	Primary: surgery; recurrence: radiotherapy + surgery	Primary site: overexpression of MDM2, DDR2; recurrent site: SDHC, TSHR	Increased atypical cytology; genetic alteration
L.N. Clore et al^[61]^	Female	8	Left cheek	−	+	+	Multiple surgeries; celecoxib or crizotinib	None	None
W. Djatisoesanto et al^[62]^	Male	30	Bladder: hematuria, a lump on the suprapubic area	−	Mass, Persistent hematuria	+	Surgeries; postoperative chemotherapy: doxorubicin + ifosfamide	None	Mitotic cells, high expression of Ki-67
X. He et al^[63]^	Female	60	Lung	None	None	+	Surgery	ALK rearrangement; SIPA1L3, ACE, BRPF3 missense mutations	Infiltration of the pericardium
Y. Shimodaira et al^[64]^	Male	81	Rectum	Liver	−	−	Steroid therapy; Surgery	FISH did not find ALK rearrangement	High expression of Ki-67
M. Spafford et al^[65]^	Female	29	Stomach	Liver	+	+	Surgery; crizotinib; Alectinib	None	High expression of Ki-67
X. Xu et al^[66]^	Female	43	Pelvic mass	−	+	+	Surgery	ALK-RRPB1, HAO1-ALK	None
P. Bonvini et al^[67]^	Male	19	Bladder	Lung, bone	−	+	Surgery; entrectinib	None	Circulating tumor cells
D. Kalita et al^[68]^	Female	32	Lung	Vertebral and supratentorial metastasis	+	+	Multiple surgeries; crizotinib; lorlatinib; whole brain radiation	None	Ganglion-like cells; high expression of Ki-67
H. Iwai et al^[69]^	Female	69	Left lung	Right lung	+	−	Multiple surgeries;	None	None

ALK = anaplastic lymphoma kinase.

In the absence of an identifiable molecular target, treatment decisions should be guided by tumor behavior. For tumors that are stable and asymptomatic, active surveillance with periodic monitoring of size and clinical symptoms is recommended to avoid unnecessary overtreatment.

## 4. Conclusion

Given that IMT originating from the broad ligament are exceedingly rare, the case reported here demonstrates distinct singularity, and its lineage of differentiation remains unresolved using current diagnostic techniques. The risks of distant metastasis and recurrence in ALK-negative patients are yet to be clearly defined, and the effectiveness of adjuvant therapies – including nonsteroidal anti-inflammatory drugs (NSAIDs), radiotherapy, chemotherapy, and targeted therapies – requires further investigation to establish their clinical value. Looking ahead, the broad application of molecular biology techniques in tumor research is expected to facilitate the discovery of novel subtypes with specific molecular alterations within mesenchymal tumors, thereby laying a robust foundation for precision diagnosis and treatment.

## Author contributions

**Conceptualization:** Hongjing Wang.

**Data curation:** Huihui Guo.

**Formal analysis:** Huihui Guo, Hongjing Wang.

**Investigation:** Huihui Guo, Hongjing Wang.

**Methodology:** Huihui Guo, Hongjing Wang.

**Project administration:** Huihui Guo, Hongjing Wang.

**Supervision:** Hongjing Wang.

**Validation:** Huihui Guo, Hongjing Wang.

**Visualization:** Hongjing Wang.

**Writing – original draft:** Huihui Guo, Hongjing Wang.

**Writing – review & editing:** Huihui Guo, Hongjing Wang.
